# The Food Environment Around Primary Schools in a Diverse Urban Area in the Netherlands: Linking Fast-Food Density and Proximity to Neighbourhood Disadvantage and Childhood Overweight Prevalence

**DOI:** 10.3389/fpubh.2022.838355

**Published:** 2022-04-06

**Authors:** Bente A. Smagge, Laura A. van der Velde, Jessica C. Kiefte-de Jong

**Affiliations:** Department of Public Health and Primary Care/LUMC-Campus the Hague, Leiden University Medical Center, Leiden University, The Hague, Netherlands

**Keywords:** primary school, food environment, fast-food, neighbourhood disadvantage, childhood overweight

## Abstract

In the Netherlands, the neighbourhood food environment has received little attention in initiatives to combat overweight/obesity. This study maps the food environment around primary schools in The Hague, The Netherlands, and examines associations between neighbourhood disadvantage, the school food environment and childhood overweight using Geographic Information Systems (GIS). Linear regression analyses were performed to test the association between schools' disadvantage scores (proxy for neighbourhood disadvantage) and relative fast-food density within 400 m and 1000 m and fast-food proximity. Univariable and multivariable linear regression analyses were used to test the association between the school food environment and overweight prevalence among children in the respective sub-district in which the schools is found. Multivariable analyses were adjusted for the schools' disadvantage scores. Results show that fast-food outlets were available around most primary schools. Schools in disadvantaged neighbourhoods were closer to and surrounded by a higher number of fast-food restaurants, grillrooms and kebab shops. On the sub-district level, the density of such fast-food outlets was associated with overweight prevalence among children. These findings highlight the importance of national and local policies to improve the food environment, particularly in disadvantaged neighbourhoods.

## Introduction

Overweight and obesity are increasingly prevalent among children (1, 2). Overweight/obese children are likely to remain overweight/obese as adults and accumulate the risks of developing non-communicable diseases posed by being overweight or obese throughout their lives (1). Due to both stigmatisation and biological mechanisms, overweight and obesity also negatively affect children's education, career, social life and psychological well-being (3, 4).

Childhood overweight/obesity does not have a single cause, nor is there a single intervention that can tackle it. Personal characteristics play a role, but many children also live in an obesogenic environment (5, 6). A substantial part of these environmental influences is food-related and encourages excessive caloric intake. Much research has been done on the influence of the neighbourhood food environment on eating and drinking habits and weight status, but results are inconsistent (7–10).

As in other places around the world, overweight prevalence is not evenly distributed among the underage population of The Hague, The Netherlands (8, 11). Disproportionally high percentages of children living in sub-districts with low socio-economic status and children from non-Western ethnic backgrounds are overweight or obese (11). This unequal distribution is not fully explained by household income and parental educational level and suggests that other environmental factors at the neighbourhood level may influence the development of excessive weight in children. A key factor at the neighbourhood level that could contribute to the unequal distribution of overweight/obesity prevalence is the food environment, particularly the spatial availability and accessibility of fast-food (8, 9, 12). Children's neighbourhood food environment includes their residential environment and the food environment in places they frequent, particularly around their school (10, 13). For Dutch primary school children, the school food environment approximates children's neighbourhood food environment as a whole because most children attend primary school close to home. Hence, the school food environment represents the environment where they spend the vast majority of their time. While primary school-aged children receive most food from their parents to bring to school (schools rarely provide meals), some do purchase food themselves before and/or after school (14). An unhealthy food environment may also invite and normalise unhealthy food preferences or choices. This is not unique to children, but children are especially sensitive to food cues (15, 16).

Previous research into the association between neighbourhood disadvantage and the food environment has shown that fast-food restaurants are more prevalent – in general and around schools – in socio-economically disadvantaged areas compared to non-disadvantaged areas (7, 17, 18). For other types of food outlets, the relationship with neighbourhood disadvantage is ambiguous (7). Most evidence for a link between neighbourhood disadvantage and fast-food availability comes from North America. In the Netherlands, however, the number of fast-food providers within five minutes from secondary schools also appears to be higher in low-income neighbourhoods, although overall healthiness of food providers did not differ by neighbourhood socioeconomic status (5, 7, 19).

Based on these findings, we hypothesise that there are more fast-food outlets around primary schools in disadvantaged neighbourhoods compared to other neighbourhoods in The Hague and that the large number of fast-food outlets contributes to the disproportionately high prevalence of overweight/obesity in The Hague's disadvantaged sub-districts. Although evidence of a differential effect of the food environment on dietary behaviour by socioeconomic status is limited, children and parents living in (highly) disadvantaged neighbourhoods may be more prone to buy quick and cheap food at fast-food outlets upon exposure to such outlets (20). Hence, we hypothesised that the higher the level of disadvantage, the stronger the association of the food environment with childhood overweight prevalence.

In the Netherlands in 2019, 13.2% of children aged 4–17 were overweight and 2.1% were obese (2). Recognising the severity of the issue and long-lasting impact on health and well-being, policymakers are dedicated to combat overweight and obesity, particularly among children, yet the food environment has not been extensively addressed in the Netherlands. Therefore, the aims of this study were 1) to map the food environment around primary schools in The Hague, The Netherlands; 2) to test the associations between neighbourhood disadvantage and measures of fast-food availability around primary schools; and 3) to examine the association between the school food environment and overweight prevalence among children and to what extent this is modified by neighbourhood disadvantage.

## Methods

### Study Context

**Figure 1:**
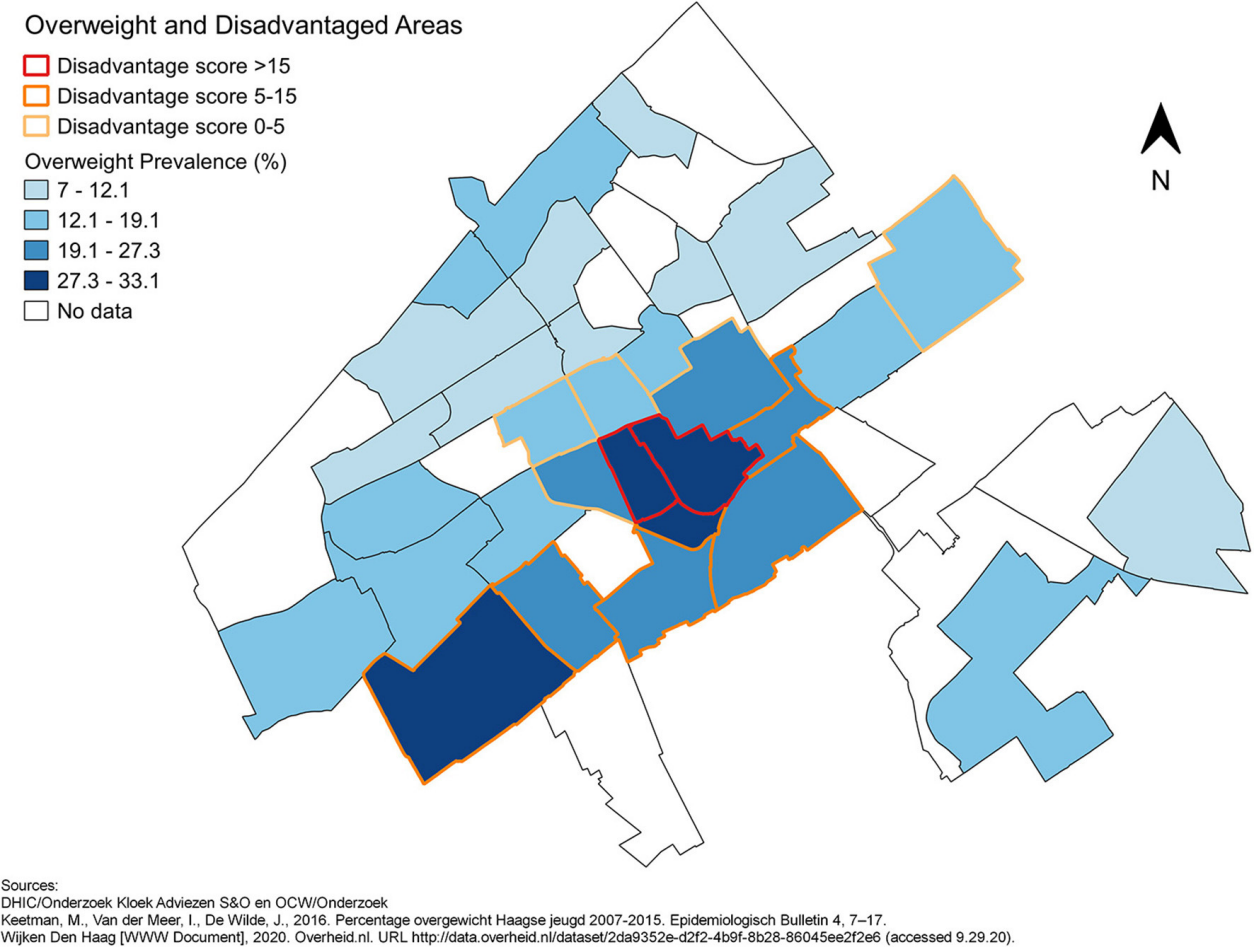
The prevalence of overweight per sub-district and the disadvantaged sub-districts in The Hague in 2015.

This cross-sectional study focussed on the Dutch city The Hague. The Hague is a city of around half a million people characterised by high levels of urbanisation and socioeconomic and ethnic diversity [Table S1, (21)]. Descriptive data about the number of inhabitants, age distribution, percentage of inhabitants with a migration background and disadvantage per sub-district were obtained from The Hague in Numbers [Den Haag in Cijfers (DHIC)] [Table S1, (21)]. The degree of disadvantage per sub-district was presented using the Disadvantage Index (22). Total scores below 0 indicate no disadvantage, while scores above 0 indicate (some form of) disadvantage. In the commonly used classification system, scores of 5–15 and scores >15 constitute the disadvantaged classes (22). The **overweight prevalence** data used for this study were collected during routine preventive health surveys among children during which weight and height are measured by trained healthcare workers (23). Weight categories are determined based on height, age and sex using Cole and Lobstein's (2012) cut-off values and adapted cut-offs for youth of South Asian ethnicity who have a different body composition (23, 24). With a participation rate of over 80% and appropriate age-adjustment, these data are considered representative of The Hague's child and youth population (23). The individual-level survey measurements were not available for this study. The sub-district level Disadvantage Index was combined with data on childhood overweight prevalence per sub-district from the youth healthcare services (in Dutch: Jeugdgezondheidszorg) in a choropleth map (Figure 1).

#### Mapping the Food Environment Around Primary Schools

To map and visualise the food environment around primary schools in The Hague, the Geographic Information Systems (GIS) software QGIS (QGIS Desktop 3.14.16 Pi and 3.16.0 Hannover) was used (25). With data from OpenStreetMap, the Education Executive Agency of the Dutch Ministry of Education, Culture and Science (DUO) and Locatus, schools were geocoded based on their addresses and the sites of fast-food outlets were plotted using their XY coordinates (26–28). Schools offering special education, such as schools for handicapped children, children with behavioural and mental health disorders and children with severe learning difficulty, were excluded. Pupils often travel further to attend these schools and, therefore, the school's food environment may not reflect the neighbourhood food environment where the children spend the majority of their time. Additionally, a substantial proportion of pupils who need special education cannot visit food outlets independently or may experience the food environment in unexpected ways (29). The Locatus dataset of retailers is based on regular systematic field audits in which stores in the Netherlands are mapped and classified (30). In order to map the food environment around schools in The Hague, particularly the fast-food environment, fast-food restaurants, grillrooms and kebab shops and take-away restaurants were mapped together with primary schools, per category and per city district. Hereafter the term “fast-food restaurants” denotes the Locatus retailer category, while “fast-food outlets” refers to the totality of fast-food restaurants, grillrooms and kebab shops and take-away restaurants.

#### Variables Used for the Statistical Analyses

This study was based on secondary data. To capture the fast-food environment, relative fast-food density (FFD) and fast-food proximity (FFP) around primary schools were assessed. **Relative FFD** was calculated using the buffer function in QGIS. Relative FFD refers to the number of fast-food outlets within a certain Euclidean distance from a school as a proportion of the total amount of food retailers in this area. In the Netherlands, home-school travel is often done on foot or by bike. Therefore, for the Euclidean buffer zones, radii of 400 (FFD_400_) and 1000 (FFD_1000_) metres were used, as these distances represent acceptable walking and short biking distances (9, 31). To determine **FFP**, the distance matrix function in QGIS was used. FFP is defined as the shortest Euclidean distance from the school to the nearest fast-food outlet.

The measure of neighbourhood disadvantage used in the statistical analyses is the **schools' disadvantage score**. This is more specific to the immediate surroundings of the school than the district or sub-district level Disadvantage Index used in the descriptive data and visual analyses. Primary schools in the Netherlands receive subsidies if there is a risk of educational disadvantage among their pupils, indicated by a high disadvantage score of the school. Educational disadvantage refers to cases where a pupil's school performance is negatively influenced by an unfavourable environment (32). The environmental conditions used to predict educational disadvantage are similar to those commonly used to identify neighbourhood disadvantage and as most children attend primary school close to home, this study uses the school disadvantage score as an indicator of neighbourhood disadvantage (33). Disadvantage scores of 0 or below 0 are equalled to 0 and indicate that there is no educational disadvantage, whereas scores above 0 indicate disadvantage (32). As scores below 0 were equalled to 0, the disadvantage score is treated as a categorical variable. At schools with a positive disadvantage score, educational disadvantage is expected and, thus, these schools are considered to be located in disadvantaged neighbourhoods. Overweight prevalence per sub-district was based on data from routine preventive health surveys among children, as described earlier.

#### Visualisation and Spatial Analysis

To visualise the potential link between disadvantage at the sub-district level and the food environment surrounding primary schools, maps of The Hague showing the locations of primary schools and the disadvantaged sub-districts were made. The schools appear in two colours depending on whether the relative FFD_400_ or FFD_1000_ or FFP is above or below average: yellow represents relative FFD above average or FFP below average, whereas purple represents relative FFD below average or FFP above average (**Figures 3**–**5**).

#### Statistical Analyses

Descriptive statistics were used to describe the school food environment and Welch's *t*-Tests were conducted to compare the school food environment in disadvantaged and non-disadvantaged sub-districts. Linear regression analyses were used to test whether the schools' disadvantage score (as a proxy for neighbourhood disadvantage) was associated with relative FFD_400_, relative FFD_1000_ and FFP. Analyses were performed using the disadvantage score as a dichotomous variable (i.e., not disadvantaged [(score = 0) and disadvantaged (score > 0)] and as a categorical variable (i.e., six categories, ranging from no to very high disadvantage). As the distribution of relative FFD_400_ and FFP were positively skewed, these variables were natural log transformed (after adding 1 to the relative FFD_400_ values to account for real zeros in the data) before regression. The number of pupils per school was controlled for, as in areas with a high number of residents, the number of food providers is likely to be relatively high. Analyses were performed with all fast-food outlets together and stratified by type: fast-food restaurants, grillrooms and kebab shops and take-away restaurants. Non-linearity was evaluated by testing a quadratic term in the regression model.

To test the association between the school food environment and the prevalence of overweight among children in the respective sub-districts in which schools are located, univariable and multivariable linear regression analyses were used. Analyses were adjusted for the schools' disadvantage score, as a proxy for neighbourhood disadvantage. Regression analyses were conducted with relative FFD and FFP of all fast-food outlets and stratified by type of fast-food outlet. Non-linearity was evaluated by using a quadratic term.

To assess whether the association between the school food environment and overweight prevalence among children was modified by the level of neighbourhood disadvantage, univariable regressions were run separately for schools with no, low, medium and high disadvantage scores. The latter three categories were obtained by disaggregating schools with a positive disadvantage score into tertiles. Potential effect modification was further assessed by including an interaction term between the disadvantage level and the food environment variables. Statistical significance was defined as *p* < 0.05 throughout the analyses. Statistical analyses were conducted in R Studio (R 4.0.3) (34).

## Results

Childhood overweight prevalence per sub-district of The Hague ranged from 7.0 to 33.1%. Childhood overweight prevalence was highest in the more disadvantaged sub-districts (Figure 1).

**Figure 2:**
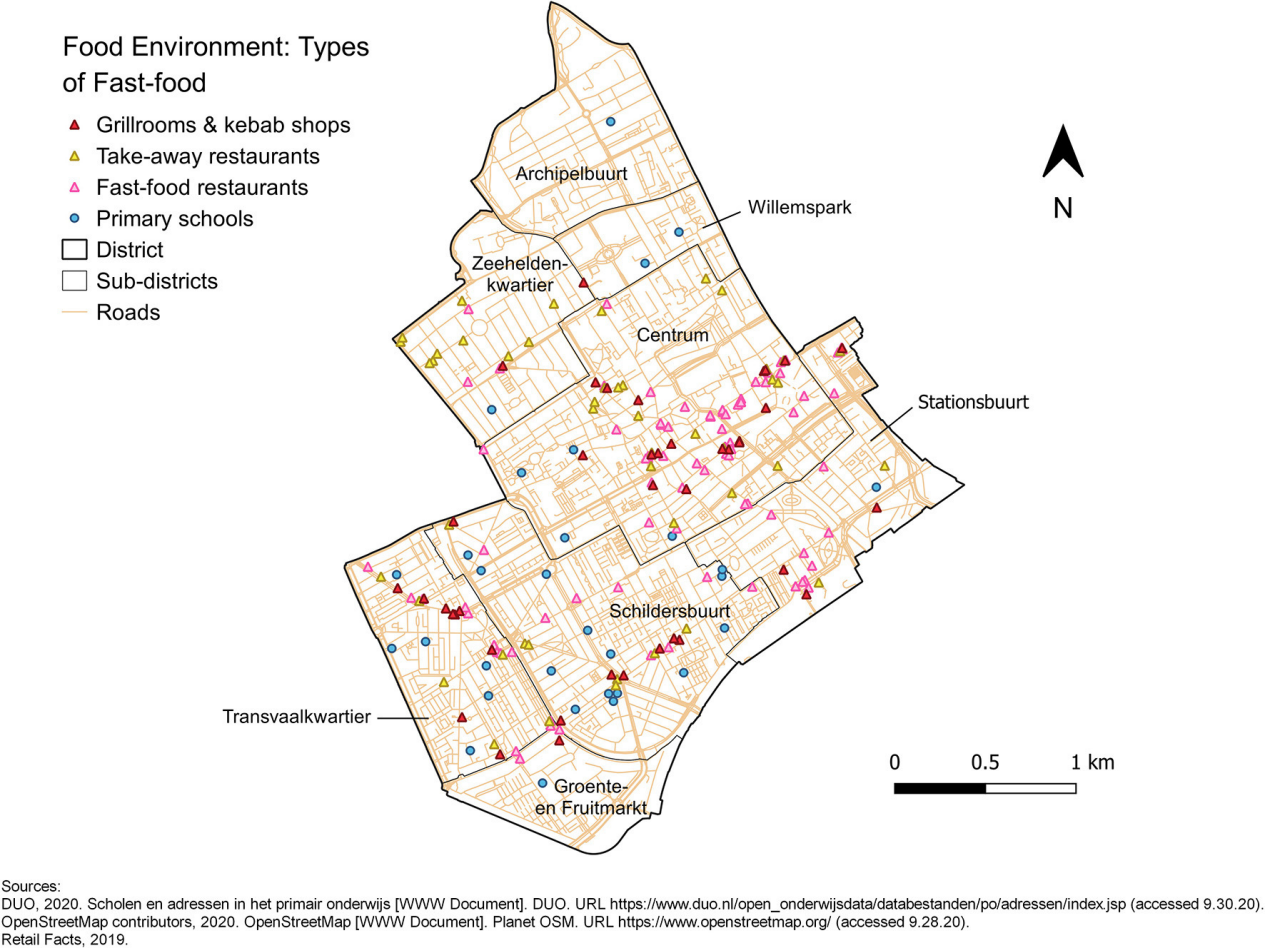
Grillrooms and kebab shops, take-away restaurants and fast-food restaurants around primary schools in the district Centrum.

Of the primary schools in The Hague, *n* = 135 included data on the school disadvantage scoreand the number of pupils. Overall, the maps showed that most schools were located nearby one or more fast-food outlet. The mean relative FFDs and FFP confirmed that fast-food was readily available around primary schools, with fast-food outlets making up around 20% of the food retailers within 400 or 1000 m from the school and the mean distance to the closest one being 277.55 m (Table 1). However, some schools also had no fast-food outlet(s) within 400 m or 1000 m. Visualising the fast-food environment around primary schools showed an unequal distribution of fast-food outlets throughout The Hague. Figure 2 shows the locations of schools and fast-food outlets in the city centre and complementary maps by district are available as Supplementary Material. Fast-food outlets were present in considerably higher numbers in some sub-districts compared to others and clusters were observed (Figure 2, Supplementary Material). Especially in and around the city centre, the number of both fast-food outlets and schools was notably high (Figure 2). Other clusters of fast-food outlets, such as those near the beach, had fewer schools nearby. Overall, fast-food restaurants were most plentiful (*n* = 253) in the city, followed by take-away restaurants (*n* = 145) and grillrooms and kebab shops (*n* = 86) (maps by category of fast-food retailer are available as Supplementary Material).

**Table 1:** Descriptive statistics of the school food environment in The Hague and results of the Welch's *t*-Tests to compare the school food environment in disadvantaged and other sub-districts.

	**City-wide**	**Disadvantaged sub-districts**	**Other sub-districts**	***t*-Test^**a**^**
Number of primary schools	135	34	101	
	*Mean (SD)*			*p-value*
Relative FFD_400_ ^b^	18.41% (15.62)	20.30% (6.45)	17.78% (17.55)	*p* = 0.23
Relative FFD_1000_ ^c^	20.10% (7.23)	20.31% (3.20)	20.03% (8.12)	*p* = 0.78
FFP^d^	277.55 m (230.67)	159.84 m (81.20)	317.17 m (249.28)	*p* <0.001

***p < 0.001, **p < 0.01, *p < 0.05, ° p < 0.01.

a *Two-tailed Welch's t-Test to test the statistical significance of a difference in means between the FFDs and FFP around primary schools located in disadvantaged sub-districts and other sub-districts of The Hague. The alternative hypotheses are that there are no true differences between the mean relative FFD_400_, mean relative FFD_1000_ or mean FFP in schools in disadvantaged sub-districts compared to schools in other sub-districts*.

b *Relative FFD_400_ refers to the number of fast-food outlets within 400 m (Euclidean distance) from a school as a proportion of the total amount of food retailers in this area*.

c *Relative FFD_1000_ refers the number of fast-food outlets within 1000 m (Euclidean distance) from a school as a proportion of the total amount of food retailers in this area*.

d *FFP is the shortest Euclidean distance from the school to the nearest fast-food outlet*.

**Figure 3:**
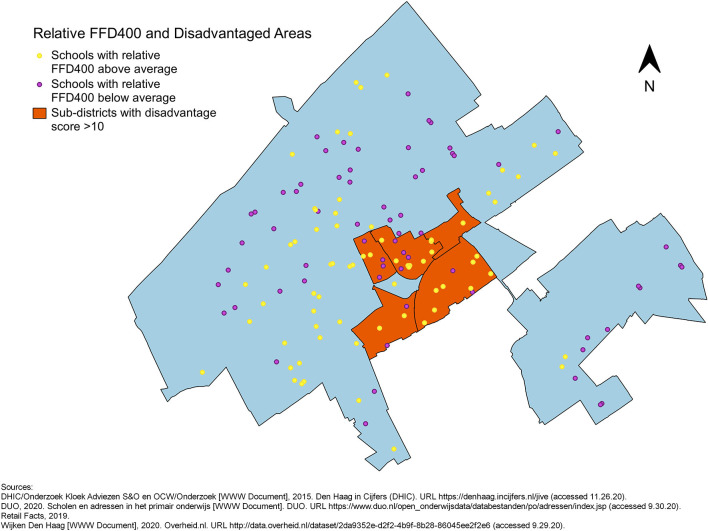
Primary schools in The Hague, plotted in different colours to indicate whether the relative FFD_400_ around them is above or below average, against an outline of city with the five disadvantaged sub-districts.

**Figure 4:**
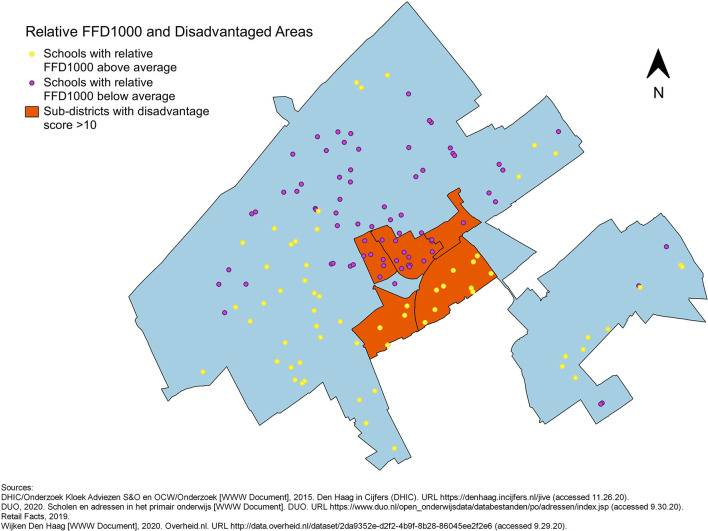
Primary schools in The Hague, plotted in different colours to indicate whether the relative FFD_1000_ around them is above or below average, against an outline of city with the five disadvantaged sub-districts.

**Figure 5:**
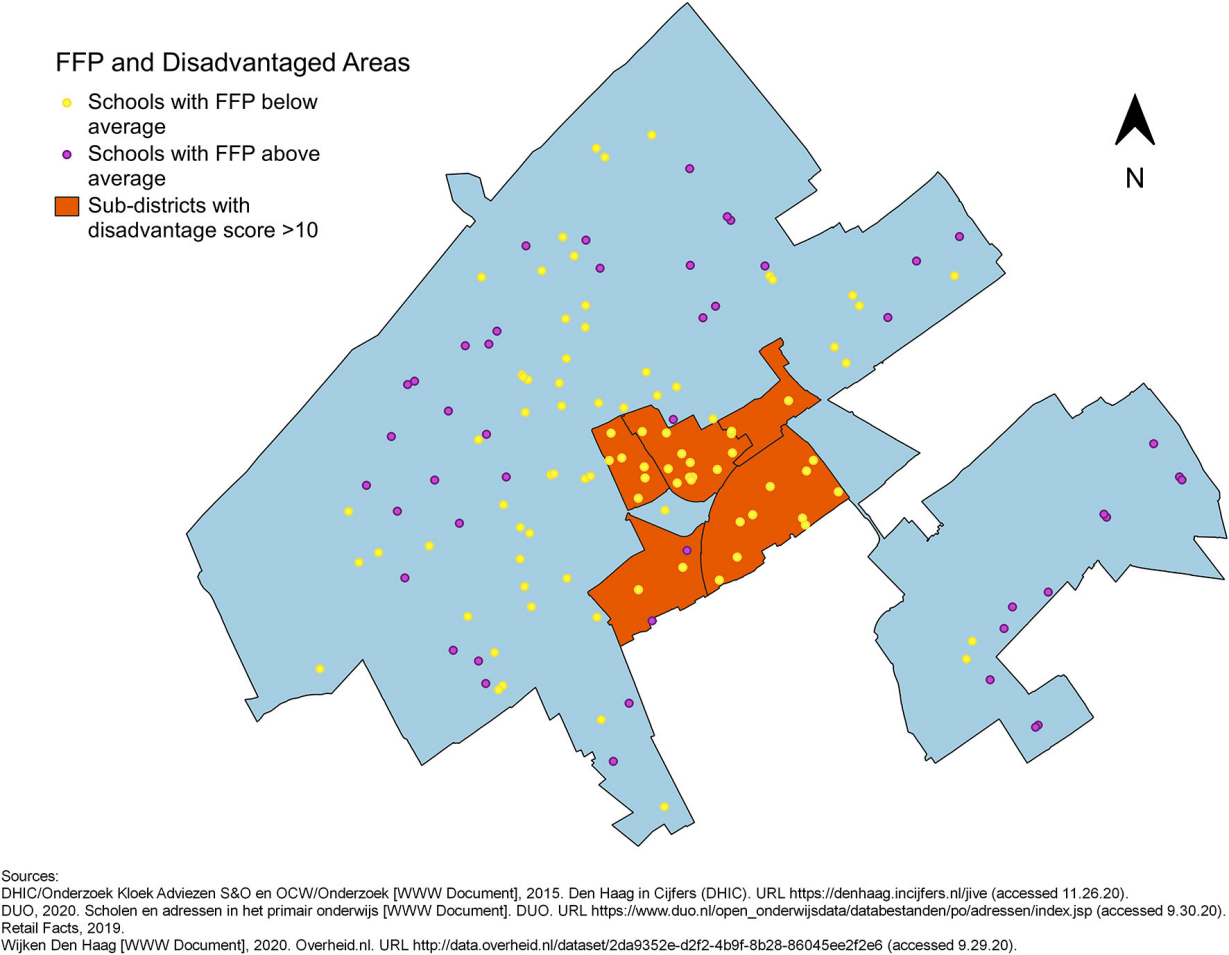
Primary schools in The Hague, plotted in different colours to indicate whether their FFP is above or below average, against an outline of city with the five disadvantaged sub-districts.

No evident pattern was observed in the maps of the distribution of schools with relative FFDs below or above average across disadvantaged and non-disadvantaged sub-districts identified by the Disadvantage Index. Many schools with high relative FFD_400_ around them were located in non-disadvantaged areas (Figure 3). The relative FFD_1000_ was high for all schools in two disadvantaged sub-districts (Laakkwartier en Spoorwijk and Moerwijk), but low in the other disadvantaged sub-districts. The proportion of schools with relative FFD_1000_ above the mean appeared quite equal in disadvantaged and non-disadvantaged areas (Figure 4). The FFP was below average for all but two schools in disadvantaged areas, but also for many schools elsewhere in the city (Figure 5). However, while the mean relative FFDs did not differ significantly between disadvantaged and other sub-districts, the mean FFP was significantly smaller in disadvantaged sub-districts compared to other sub-districts (Table 1).

Results of the linear regression analyses showed that the schools' disadvantage score, dichotomously and in six categories, was associated with higher relative FFD_400_ [β = 1.20; 95% CI (0.78, 1.62) and β = 0.34; 95% CI (0.21, 0.47)], higher FFD_1000_ [β = 3.05; 95% CI (0.48, 5.62) and β = 1.09; 95% CI (0.33, 1.84)] and lower FFP [β = −0.56; 95% CI (−0.81, −0.30) and β = −0.18; 95% CI (−0.25, −0.10)] of fast-food outlets (Table 2). The results stratified by fast-food outlet type showed that for fast-food restaurants separately, the disadvantage score was associated with higher relative FFD_400_ and lower FFP. Only the disadvantage score in six categories was associated with higher relative FFD_1000_. The disadvantage score was also associated with the relative density and proximity of grillrooms and kebab shops. The associations between disadvantage and the relative FFDs and FFP were stronger for grillrooms and kebab shops than for fast-food restaurants. For take-away restaurants, only the associations between the disadvantage score, dichotomously and in six categories, and FFP and the association between the disadvantage score in six categories and relative FFD_1000_ were significant. The latter association was not in the expected direction, i.e., a higher disadvantage score was associated with lower relative FFD_1000_ (Table 2).

**Table 2:** Associations between neighbourhood disadvantage and the food environment.

	**Association with the food environment** ^**a**^		
	***Estimated impact** **β** **(95% confidence interval)***		
	**Relative FFD_**400**_ ^**b**^**	**Relative FFD_**1000**_ ^**c**^**	**FFP^**d**^**
**All fast-food outlets**
Disadvantage score (dichotomous)	1.20^***^ (0.78, 1.62)	3.05* (0.48, 5.62)	−0.56^***^ (−0.81, −0.30)
Disadvantage score (six levels)	0.34^***^ (0.21, 0.47)	1.09^**^ (0.33, 1.84)	−0.18^***^ (−0.25, −0.10)
**Fast-food restaurants**
Disadvantage score (dichotomous)	1.11^***^ (0.75, 1.47)	1.59 (−0.49, 3.68)	−0.46^***^ (−0.70, −0.23)
Disadvantage score (six levels)	0.29^***^ (0.18, 0.40)	0.70^*^ (0.09, 1.31)	−0.15^***^ (−0.21, −0.08)
**Grillrooms and kebab shops**
Disadvantage score (dichotomous)	1.18^***^ (0.86, 1.49)	2.43^***^ (1.33, 3.53)	−0.96^***^ (−1.30, −0.62)
Disadvantage score (six levels)	0.40^***^ (0.31, 0.49)	0.93^***^ (0.62, 1.24)	−0.37^***^ (−0.46, −0.28)
**Take-away restaurants**
Disadvantage score (dichotomous)	0.25 (−0.14, 0.64)	−0.98 (−2.73, 0.77)	−0.34^*^ (−0.63, −0.04)
Disadvantage score (six levels)	0.08 (−0.04, 0.20)	−0.55^*^ (−1.06, −0.03)	−0.11^*^ (−0.19, −0.02)

*** *p < 0.001*,

** *p < 0.01*,

* *p < 0.05, ° p < 0.01*.

a *Linear regression was used to test the association between neighbourhood disadvantage and the food environment, controlling for the number of pupils per school. Analyses were run both with disadvantage score as a dichotomous variable (neighbourhood disadvantage compared to reference category no neighbourhood disadvantage) and with disadvantage score as a categorical variable (six levels)*.

b *The variable relative FFD_400_ was transformed using the expression ln(X + 1). Relative FFD_400_ refers to the number of fast-food outlets within 400 m (Euclidean distance) from a school as a proportion of the total amount of food retailers in this area*.

c *Relative FFD_1000_ refers the number of fast-food outlets within 1000 m (Euclidean distance) from a school as a proportion of the total amount of food retailers in this area*.

d *The variable FFP was transformed using the expression ln(X). FFP is the shortest Euclidean distance from the school to the nearest fast-food outlet*.

Higher relative FFD_400_ and FFD_1000_ were associated with higher prevalence of overweight among children in the sub-district [β = 2.74; 95% CI (1.73, 3.76) and β = 1.09; 95% CI (0.55, 1.64), respectively], while greater distance to fast-food outlets was also associated with higher overweight prevalence [β = 25.33; 95% CI (7.70, 42.96)]. Controlling for the disadvantage level slightly weakened these associations, but associations with relative FFD_400_ [β = 1.16; 95% CI (0.46, 1.86)] and FFD_1000_ [β = 0.46; 95% CI (0.10, 0.82)] remained significant (Table 3). Separate analyses per fast-food category showed that higher relative densities of fast-food restaurants and grillrooms and kebab shops were associated with higher overweight prevalence among children. For take-away restaurants, higher relative FFD_400_ was associated with higher, and relative FFD_1000_ with lower, overweight prevalence. Greater distance to fast-food restaurants and take-away restaurants was associated with higher overweight prevalence among children, while greater distance to grillrooms and kebab shops was associated with lower overweight prevalence. When controlling for neighbourhood disadvantage level, the stratified results were in similar directions. However, the associations with overweight prevalence of relative FFD_1000_ of take-away restaurants, FPP of all fast-food outlets and of fast-food restaurants and take-away restaurants, specifically, were no longer significant (Table 3).

**Table 3:** Associations of neighbourhood disadvantage and the food environment with overweight among children in the sub-district that the school is located in.

	**Association with overweight among children in the sub-district**	
	***β** **(95% confidence interval)***	
	**Univariable**	**Multivariable^**a**^**
**Relative FFD**_**400**_ ^**b**^		
All fast-food outlets	2.74^***^ (1.73, 3.76)	1.16^**^ (0.46, 1.86)
Fast-food restaurants	7.71^***^^c^ (4.82, 10.61)	1.54^***^ (0.76, 2.33)
Grillrooms & kebab shops	5.11^***^ (4.01, 6.22)	2.15^***^ (1.15, 3.15)
Take-away restaurants	10.57***^c^ (6.21, 14.94)	3.69^*^^c^ (0.67, 6.71)
**Relative FFD**_**1000**_ ^**d**^		
All fast-food outlets	1.09^***^^c^ (0.55, 1.64)	0.46^*^^c^ (0.10, 0.82)
Fast-food restaurants	1.15^***^^c^ (0.66, 1.64)	0.45^**^^c^ (0.11, 0.78)
Grillrooms & kebab shops	3.39^***^^c^ (2.34, 4.45)	2.01^***^^c^ (1.13, 2.89)
Take-away restaurants	−1.07^***^^c^ (−1.67, −0.47)	−0.18° (−0.36, 0.005)
**FFP** ^**e**^		
All fast-food outlets	25.33^**^^c^ (7.70, 42.96)	11.54°^c^ (−0.17, 23.25)
Fast-food restaurants	33.38^**^^c^ (12.58, 54.18)	13.91°^c^ (−0.04, 27.86)
Grillrooms & kebab shops	−4.48^***^ (−5.60, −3.35)	−1.60^**^ (−2.56, −0.63)
Take-away restaurants	20.64^*^^c^ (3.11, 38.17)	−0.26 (−1.34, 0.82)

*** *p < 0.01*,

** *p < 0.05*,

* *p < 0.1, ° p < 0.001*.

a *The multivariable regressions were adjusted for the neighbourhood disadvantage level around schools (based on the schools' disadvantage scores)*.

b *The variable relative FFD_400_ was transformed using the expression ln(X + 1). Relative FFD_400_ refers to the number of fast-food outlets within 400 m (Euclidean distance) from a school as a proportion of the total amount of food retailers in this area*.

c *This effect was better estimated by including a quadratic term in the regression*.

d *Relative FFD_1000_ refers the number of fast-food outlets within 1000 m (Euclidean distance) from a school as a proportion of the total amount of food retailers in this area*.

e *The variable FFP was transformed using the expression ln(X). FFP is the shortest Euclidean distance from the school to the nearest fast-food outlet*.

No significant interaction was observed between the disadvantage score and the food environment in the association between the food environment and childhood overweight (Table 4).

**Table 4:** Associations between the food environment and overweight prevalence among children in the sub-district that the school is located in and the interaction between the food environment and neighbourhood disadvantage in these associations.

	**Association with overweight among children in the sub-district** ^**a**^				***P*-value interaction term**
	***β** **(95% confidence interval)***				
	**No disadvantage schools**	**Low disadvantage score schools**	**Medium disadvantage score schools**	**High disadvantage score schools**	
Relative FFD_400_ ^b^	−2.38°^c^ (−4.81, 0.06)	1.36 (−0.36, 3.09)	4.95*** (2.33, 7.56)	0.41 (−1.38, 2.19)	*p* = 0.10
Relative FFD_1000_	0.15* (0.03, 0.28)	0.008 (−0.25, 0.27)	3.42**^c^ (1.11, 5.73)	−0.18 (−0.47, 0.11)	*p* = 0.26
FFP^d^	0.42 (−0.92, 1.75)	−0.77 (−3.18, 1.65)	79.80**^c^ (22.62, 136.98)	−0.88 (−3.50, 1.74)	*p* = 0.36

^***^p < 0.01, ^**^p < 0.05, ^*^p < 0.1, ° p < 0.001.

a *Separate regressions were run with schools with disadvantage score = 0 and schools with low, medium and high disadvantage (disaggregation of schools with disadvantage score > 0 into tertiles). The p-value of the interaction term refers to the interaction between a food environment variable and the disadvantage score category (no, low, medium or high disadvantage)*.

b *The variable relative FFD_400_ was transformed using the expression ln(X + 1)*.

c *This effect was better estimated by including a quadratic term in the regression*.

d *The variable FFP was transformed using the expression ln(X)*.

## Discussion

The results of this study indicate that the density of fast-food restaurants, grillrooms and kebab shops around primary schools is higher and the distance between schools and such fast-food outlets is smaller in disadvantaged neighbourhoods compared to non-disadvantaged neighbourhoods. The findings further suggest that greater density of fast-food outlets is associated with greater overweight prevalence among children on the sub-district level.

Our findings indicate that higher disadvantage scores of primary schools, representing neighbourhood disadvantage, are associated with higher relative fast-food density and closer fast-food proximity. This is in line with previous research (7, 17, 18). However, few studies have shown such associations in the Netherlands (5, 7, 17–19). The majority of studies have been conducted in the United States, where ethnic and socioeconomic segregation are more pronounced (7).

The visualisations in this study show that in disadvantaged sub-districts the FFP from nearly all schools was below average (i.e., fast-food outlets were generally located closer to these schools), whereas non-disadvantaged sub-districts contained schools with both high and low FFP. Schools with high relative FFDs were also found in both disadvantaged and non-disadvantaged sub-districts. Thus, although this study supports previous findings that link area disadvantage to fast-food availability/accessibility, it emphasises that an unhealthy food environment surrounding primary schools is also a concern outside disadvantaged areas. The apparent lack of association between disadvantage and relative FFD in the visual analysis could be explained by the fact that there are schools in the non-disadvantaged sub-districts with a Disadvantage Score above 0, indicating they are in a disadvantaged neighbourhood. This insight was obtained by our visual exploration of the data prior to the analyses. It suggests that there are localised differences in neighbourhood disadvantage, which are associated with relative FFD, but not captured by the sub-district level disadvantage classification. These localised differences may be explained by residential and school segregation, for example (35).

Stratification by type of fast-food outlet showed an association between schools' disadvantage score and the food environment for fast-food restaurants and grillrooms and kebab restaurants, but this association was less evident for take-away restaurants. An explanation could be that take-away restaurants may include more expensive take-away and delivery restaurants (e.g. sushi) (27). Additionally, spatial observations may be misleading if restaurants rely more on delivery than take-away. A greater presence of grillrooms and kebab restaurants in disadvantaged areas might be expected because more people with a migration background live there who generally consume these foods more often or own the grillrooms and kebab restaurants (21, 36). However, our findings show that also other fast-food outlets are significantly closer to schools and more prevalent around schools with higher disadvantage scores.

One explanation that may contribute to the uneven distribution of fast-food restaurants, grillrooms and kebab shops is that citizens living in disadvantaged neighbourhoods may be less represented in local politics and that community-participation in political decision-making regarding the food environment in these neighbourhoods may be lower (37). However, also time pressure, financial concerns, stress and mental health issues and lower health consciousness among inhabitants of disadvantaged neighbourhoods may maintain a high fast-food demand (38, 39). This would create a vicious circle whereby demand raises supply and availability stimulates buying fast-food. A high demand for fast-food may also result from the more central, hence potentially more busy, location of schools in disadvantaged neighbourhoods. However, based on our observations, primary schools in The Hague are usually located in residential areas that are not very busy with people passing by even when located close to a more central location (40).

The results of this study suggest that the disparities in the food environment between disadvantaged and non-disadvantaged neighbourhoods may contribute to the higher overweight prevalence among children in disadvantaged sub-districts. High relative FFD_400_ and FFD_1000_ were significantly associated with high childhood overweight prevalence in the sub-district that the school is situated in. However, as elaborated upon below, further research into these associations is required to confirm our results. Stratification by type of fast-food outlet showed that the associations between the relative density of fast-food outlets and overweight prevalence were due to the distribution of fast-food restaurants and grill and kebab shops. Multiple associations with the relative density and proximity of take-away restaurants were in the opposite direction or non-significant. This may be because take-away restaurants are not necessarily unhealthy and generally more expensive than fast-food restaurants and grillrooms and kebab shops. Additionally, some outlets in the take-away category primarily deliver (27). The unexpected associations between higher FFP and higher childhood overweight for fast-food outlets generally and for fast-food restaurants may be explained by a non-linear relationship, but were no longer significant after controlling for schools' disadvantage levels. For grillrooms and kebab shops, the association remained significant in the opposite direction, in line with our hypothesis.

The food environment can influence consumption and weight in numerous ways (9, 41). The model by Glanz et al. divides the food environment into the information, occupational, community and consumer nutritional environments (41). Particularly differences in the community (i.e., the amount, type and location of food retailers) and the consumer (i.e., available choice, food placement and promotion, nutritional information, price and freshness inside stores and restaurants) environments could create local differences in consumption patterns and contribute to spatial inequalities in overweight prevalence.

More specifically, the food environment around primary schools, serving as a proxy for children's neighbourhood environment, could affect consumption and weight because parents/caretakers can easily purchase food on the way to and from school, possibly because their children ask for it. Children themselves too, especially those in higher grades of primary school (aged ± 10–12) can visit nearby food outlets, particularly if their parents are not around (14). A less direct influence of the food environment on eating habits is through food cues. Seeing and smelling food stimulates buying and eating it. Children are generally more sensitive to such cues than adults and also prone to copying their peers (15, 16, 42). Depending on the type of food outlets around schools and what customers buy, their presence, therefore, may encourage and normalise unhealthy eating habits for children and their parents/caretakers (15). Additionally, if the demand is high around schools and food outlets are numerous, competition between food outlets could lower food prices (15, 43).

One possible reason why we did not find stronger associations between the food environment and childhood overweight prevalence among schools with higher levels of disadvantage is because fast-food could also be unaffordable for people facing severe disadvantage. Another explanation may be that our findings can be attributed to heterogeneity amongst food purchasers. Even in areas of low socioeconomic status, some people buy food impulsively, others plan their purchases, some enjoy grocery shopping, while others hardly take time to buy food (44). The capacity to make healthy choices is also determined by an individual's level of executive control. The circumstances of poverty or low socioeconomic status reduce executive control by demanding a lot of mental energy, but they do not influence everyone to the same degree (45, 46). Therefore, people are not equally affected by the food environment.

Study strengths include stratification by fast-food type and combining visual/spatial and statistical analyses. The maps visualise the uneven distribution of fast-food outlets and provide additional information alongside the statistical results, namely that an unhealthy food environment is also an issue in non-disadvantaged areas. Furthermore, this study is set in an urban area outside of the United States and one of few studies of its kind in the Netherlands, and the first that focused on primary schools. By including nearly all primary schools, selection bias was avoided. Finally, this study is based on objective measurements and validated data. The Locatus dataset was validated in 2019 through an independent field audit and was found to contain accurate data (30). The categories fast-food, grillroom/kebab and take-away/delivery of the Locatus retailer classification have previously been used to identify fast-food outlets (47).

The results of this study should be interpreted in light of its limitations. The findings concerning the associations between the food environment and overweight prevalence should be treated with caution. Firstly, the analyses could not be controlled for individual-level factors as these data were not available. Secondly, we do not have information about children not attending the surveys where height and weight were measured. However, as routine preventive youth healthcare visits have a coverage of 80%, missing data is mostly due to lack of parental consent, moving to another area or sick leave. Therefore, we expect that this missingness had no major impact on our results. Thirdly, we used FFD around schools and schools' disadvantage scores as a proxy for neighbourhood disadvantage, which are highly localised measures, whereas overweight prevalence was available at the (less precise) sub-district level. Future researchers could calculate children's Body Mass Index (anthropometrically) and conduct multivariable regressions with indicators of the neighbourhood food environment and individual control variables, like age, gender, physical activity and socioeconomic status (48). As exposure to fast-food outlets is expected to increase overweight/obesity prevalence by promoting fast-food purchasing and consumption, purchasing behaviour and dietary intake (specifically of fast-food) are key intermediaries that should be measured to understand the mechanisms through which the neighbourhood food environment influences weight and potential effect modification. This study is limited to measuring availability and accessibility of fast-food outlets, but future studies could take into account their affordability and acceptability and properties of the food products on offer as well, as these aspects are also important for food purchase and consumption behaviour (9, 49). One approach would be to qualitatively study and map experiences of the food environment of parents and primary school children as well as secondary school pupils.

Other potential limitations of this study concern the choice of indicators. Firstly, the primary school food environment is conceptualised as the neighbourhood food environment where school children spend most of their time. In previous research both Euclidean and network distances have been used to capture the food environment (50). These measures are highly correlated, but the area within a certain radius measured as the crow flies is larger than the area within the same radius measured on the road (50). This was not considered a problem as Dutch children are likely to bike, allowing them to cover larger distances more easily than children in study settings where walking rather than biking is the norm. To confirm that FFD_400_ and FFD_1000_ are appropriate indicators, interactive mapping can be used (51).

Furthermore, as the inconsistencies in the results regarding take-away restaurants allude to, there is no consensus about how fast-food should be defined, if/how food sources can be classified as healthy or unhealthy and how to measure the food environment. This study assumes that fast-food, available at fast-food restaurants, grillrooms and kebab shops and take-away restaurants, is unhealthy and contributes to the development of overweight. However, this is not necessarily so, particularly for take-away restaurants. Further, researchers use different indicators of food availability and accessibility (9, 12). This study uses both density and proximity to give a comprehensive impression of the food environment, complemented by visualisations. Relative FFD rather than absolute FFD was used. Relative FFD takes into account that high availability of unhealthy foods may be proportional to overall food availability and, thus, high in densely populated areas where the demand is high. However, the potential influences of exposure to fast-food outlets may remain even if other food providers are nearby (although they could be counterbalanced by healthier alternatives), while the association with overweight prevalence may not be visible when measuring relative FFD.

Finally, on the one hand, the use of schools' educational disadvantage score as a proxy for neighbourhood disadvantage is a strength as it matches the spatial dimensions of FFD_400_, FFD_1000_ and FFP as measures of the school food environment. On the other hand, educational disadvantage is not exactly the same as disadvantage. Nonetheless, compared with the sub-district-level Disadvantage Index, all schools in disadvantaged sub-districts have an educational disadvantage score above 0, although there are also schools with scores above 0 in non-disadvantaged sub-districts. The average educational disadvantage score among schools in disadvantaged sub-districts is higher than in non-disadvantaged sub-districts.

This study shows that many primary schools, especially those in disadvantaged neighbourhoods, are situated in an unhealthy food environment and it suggests that this is associated with childhood overweight prevalence. These findings imply that change is needed, which raises the question of who should address the problem. For the community food sub-environment, as defined in the model by Glanz et al., this is likely municipalities, whereas the consumer and information environments may be better addressed on a national level (41).

The community food environment is a local issue, but it seems unlikely that local inhabitants and/or local institutions, including schools, will actively try to influence their food environment. Laws or policies they could appeal to are also lacking, as explained below (52). Apart from resistance to extremely easily accessible hot snacks at “snack-walls” in supermarkets near schools, the school food environment has not received much attention in the Netherlands. Secondary schools make an effort to make their own cafeterias healthier and teach about healthy eating, but do not push for a healthy food environment *around* their premises (53).

Therefore, action by municipalities is desirable to change the community food environment. In other countries, local authorities have used zoning laws. Zoning laws can influence the physical neighbourhood environment by incentivising or restricting land use by certain types of food outlets (49, 54, 55). Examples include prohibiting the opening of new fast-food restaurants within 400 m from schools and limiting the number of consecutive take-away restaurants or the proportion of retail area they occupy (55–57). Such measures also be tailored to specific disadvantaged areas, as the within-city variation and the association with neighbourhood disadvantage found by this study suggest is necessary.

Although attention to the food environment is growing, zoning has not been used in the Netherlands. Municipal authorities lack the legal means to ban unhealthy food outlets from parts of their municipality based on the potential detrimental health effects of consuming food sold there (52). Such regulation is hindered by the segregation of policy areas, as health is not a sufficiently strong consideration in policy fields like area development and the (living) environment. Even under the revised Environment and Planning Act (Omgevingswet), effective as of 2022, health still is only considered if a specific aspect of the physical living environment directly harms the health of the people in the surrounding area (58). To allow municipalities to ban fast-food outlets from specific areas, the Act should be amended to encompass a wider definition of a safe and healthy living environment (52). It should, however, be noted that the impact of zoning on overweight/obesity remains questionable (55, 57). Any such policy implementation should be carefully evaluated.

Alternative ways to improve the neighbourhood food environment are by regulating what consumers encounter inside stores (i.e., the consumer environment) and food marketing (i.e., the information environment) on a national level. A recent expert evaluation of the implementation of the Dutch government's policy regarding the food environment concludes that the current body of policy is weak (59). The expert panel advises the Dutch government to impose regulations regarding the nutritional composition and pricing of food items and to prohibit marketing targeted at children of products not in the national nutrition guidelines (59).

## Conclusion

This study shows that there is reason for concern regarding the food environment that school-aged children live in. In the Dutch city The Hague, the density of fast-food restaurants, grillrooms and kebab shops around primary schools was disproportionally high and the distance from primary schools to such fast-food outlets was disproportionally small in disadvantaged neighbourhoods. The findings also suggested that an unhealthy food environment may be associated with increased overweight prevalence among children. Future research should investigate individual weight status and other individual-level variables to control for potential confounders, possibly in longitudinal studies. Furthermore, more research into the potential interaction between disadvantage and the food environment in their effect on overweight prevalence is warranted. A positive finding would reinforce the need to intervene in the food environment to reduce childhood overweight particularly in disadvantaged areas. This study supports national and especially local policies to improve the food environment and suggests that disadvantaged neighbourhoods should be specifically targeted for interventions aimed at improving the food environment.

## Data Availability Statement

Publicly available datasets were analysed in this study. This data can be found here: Locatus: https://locatus.com - DUO (school addresses): https://www.duo.nl/open_onderwijsdata/databestanden/po/adressen/index.jsp - GGD Haaglanden (overweight prevalence): https://www.ggdhaaglanden.nl/over/publicaties-en-onderzoeken/epidemiologisch-bulletin/epidemiologisch-bulletin-2016.htm - OpenStreetMap: https://www.openstreetmap.org/.

## Author Contributions

BS drafted the manuscript, conducted the analyses, and created the visual materials. All authors were involved in developing the research question and drafting the statistical analysis plan, with most input from JK-dJ. All authors have read, edited, and approved the final manuscript.

## Conflict of Interest

The authors declare that the research was conducted in the absence of any commercial or financial relationships that could be construed as a potential conflict of interest.

## Publisher's Note

All claims expressed in this article are solely those of the authors and do not necessarily represent those of their affiliated organizations, or those of the publisher, the editors and the reviewers. Any product that may be evaluated in this article, or claim that may be made by its manufacturer, is not guaranteed or endorsed by the publisher.
