# Supplementary material for: The Food Environment Around Primary Schools in a Diverse Urban Area in the Netherlands: Linking Fast-Food Density and Proximity to Neighbourhood Disadvantage and Childhood Overweight Prevalence
Source: Front Public Health. 2022 Apr 6;10:838355. doi: 10.3389/fpubh.2022.838355 (PMC9019046; doi:10.3389/fpubh.2022.838355)
Supplement: Supplementary file 1 [file Data_Sheet_1.pdf]

Table of Contents

Table S1 – Background data of The Hague per sub-district 2

Figure S1 – The Hague districts 4

Figure S2 – Grillrooms and kebab shops, take-away restaurants and fast-food restaurants around primary schools in the district Escamp 5

Figure S3 – Grillrooms and kebab shops, take-away restaurants and fast-food restaurants around primary schools in the district Haagse Hout and two adjacent sub-districts.................. 6

Figure S4 – Grillrooms and kebab shops, take-away restaurants and fast-food restaurants around primary schools in the district Laak 7

Figure S5 – Grillrooms and kebab shops, take-away restaurants and fast-food restaurants around primary schools in the district Leidschenveen-Ypenburg... 8

Figure S6 – Grillrooms and kebab shops, take-away restaurants and fast-food restaurants around primary schools in the district Loosduinen 9

Figure S7 – Grillrooms and kebab shops, take-away restaurants and fast-food restaurants around primary schools in the district Scheveningen... 10

Figure S8 – Grillrooms and kebab shops, take-away restaurants and fast-food restaurants around primary schools in the district Segbroek 11

Figure S9 – Fast-food restaurants around primary schools in The Hague 12

Figure S10 – Grillrooms and kebab shops around primary schools in The Hague.................. 13

Figure S11 – Take-away restaurants around primary schools in The Hague........................... 14

Table S1 – Background data of The Hague per sub-district.

Sub-district	Number of inhabitants (2020)	Dis-advantage Index (2015)[1]	% aged 0-19 (2020)	% aged 20-64 (2020)	% aged >65 (2020)	% with migration background (2020)	Childhood overweight (%) (2015)
Oostduinen							
Belgisch Park	8351	-9.9	21.9%	57.7%	20.5%	38.2%	10.1
Westbroekpark en Duttendel	2171	-21.3	17.4%	39.9%	42.7%	34.5%	
Benoordenhout	14347	-16.9	23.5%	50%	26.5%	41%	7.5
Archipelbuurt	6147	-16.2	18.6%	58.6%	22.8%	42.7%	7.0
Van Stolkpark en Scheveningse Bosjes	746	-21.2	23.3%	49.3%	27.3%	46.6%	
Scheveningen	18303	-6.5	18.8%	61.2%	20%	32.4%	15.7
Duindorp	5902	-3.7	23.3%	61.4%	15.3%	18.8%	17.8
Geuzen- en Statenkwartier	13987	-13.6	23.5%	57.9%	18.6%	42.4%	10.3
Zorgvliet	619		17.6%	47.2%	35.2%	46%	
Duinoord	8148	-7.7	20.2%	65.7%	14.2%	43.3%	8.6
Bomen- en Bloemenbuurt	14797	-10.8	20.5%	58.5%	20.9%	32.4%	10.0
Vogelwijk	5343	-25.9	30.5%	48.9%	20.6%	27.6%	8.3
Bohemen en Meer en Bos	4868	-11.1	12.6%	49.6%	37.8%	27.2%	7.8
Kijkduin en Ockenburgh	2385	-19.2	18.2%	48.4%	33.3%	28.6%	
Kraayenstein en Vroondaal	6808	-10.5	24%	59.2%	16.8%	30.7%	12.8
Loosduinen	18072	-7.2	20.3%	55.6%	24.1%	34.2%	16.5
Waldeck	17514	-5.3	16.2%	51.1%	32.7%	34.4%	17.3
Vruchtenbuurt	9875	-12.4	23.2%	60.3%	16.6%	26.9%	9.0
Valkenbos-kwartier	18508	1.8	19.2%	70.4%	10.4%	51.4%	17.2
Regentesse-kwartier	13893	4.9	17.7%	71.7%	10.6%	52.1%	16.7
Zeehelden-kwartier	12317	-0.1	16.3%	72.6%	11.1%	50.9%	15.0
Willemspark	1611	-10.5	14.7%	66.7%	18.6%	53.4%	
Haagse Bos	477		5.9%	50.5%	43.6%	46.1%	
Mariahoeve en Marlot	15045	2.1	18.8%	59.4%	21.8%	54.3%	19.1
Bezuidenhout	17198	-3.2	19%	68.8%	12.3%	50.9%	14.5
Stationsbuurt	12760	10.6	18.3%	73.5%	8.2%	72.6%	25.3
Centrum	20300	3.9	14%	73.4%	12.5%	58.8%	21.2
Schildersbuurt	31669	16.4	27.4%	61.8%	10.8%	91.4%	31.3
Transvaal-kwartier	16424	16.2	26.5%	63.8%	9.7%	93%	33.1
Rustenburg en Oostbroek	19116	4.6	22.5%	69.5%	8%	67.9%	25.8

Leyenburg	15190	-4.2	18.8%	63.3%	17.9%	39.9%	18.0
Bouwlust en Vrederust	29523	7.5	26.6%	57.8%	15.6%	72.4%	29.2
Morgenstond	20251	7.6	23.5%	63.8%	12.6%	70.6%	26.1
Zuiderpark	117			32.5%	66.7%	28.2%	
Moerwijk	21504	12.6	25.9%	63.7%	10.4%	76.3%	23.7
Groente- en Fruitmarkt	5228	9.9	28%	64.9%	7.1%	89.6%	29.2
Laakkwartier en Spoorwijk	43137	11.3	23.1%	68.7%	8.2%	77.7%	27.3
Binckhorst	2758		7.4%	90.8%	1.8%	71.6%	
Wateringse Veld	22393	-6.3	28.8%	60%	11.2%	47.6%	16.6
Hoornwijk	340		18.2%	70.3%	11.5%	50.9%	
Ypenburg	27172	-8.7	30.6%	61.6%	7.9%	43.2%	15.2
Forepark	125		12.8%	58.4%	28.8%	14.4%	
Leidschenveen	20896	-9.1	30.6%	60%	9.3%	42.8%	12.1

[1] The most recent available data was used for all variables except for the disadvantage index, because in the analyses disadvantage index data from 2015 was utilised in conjunction with data on overweight prevalence among children from 2015.

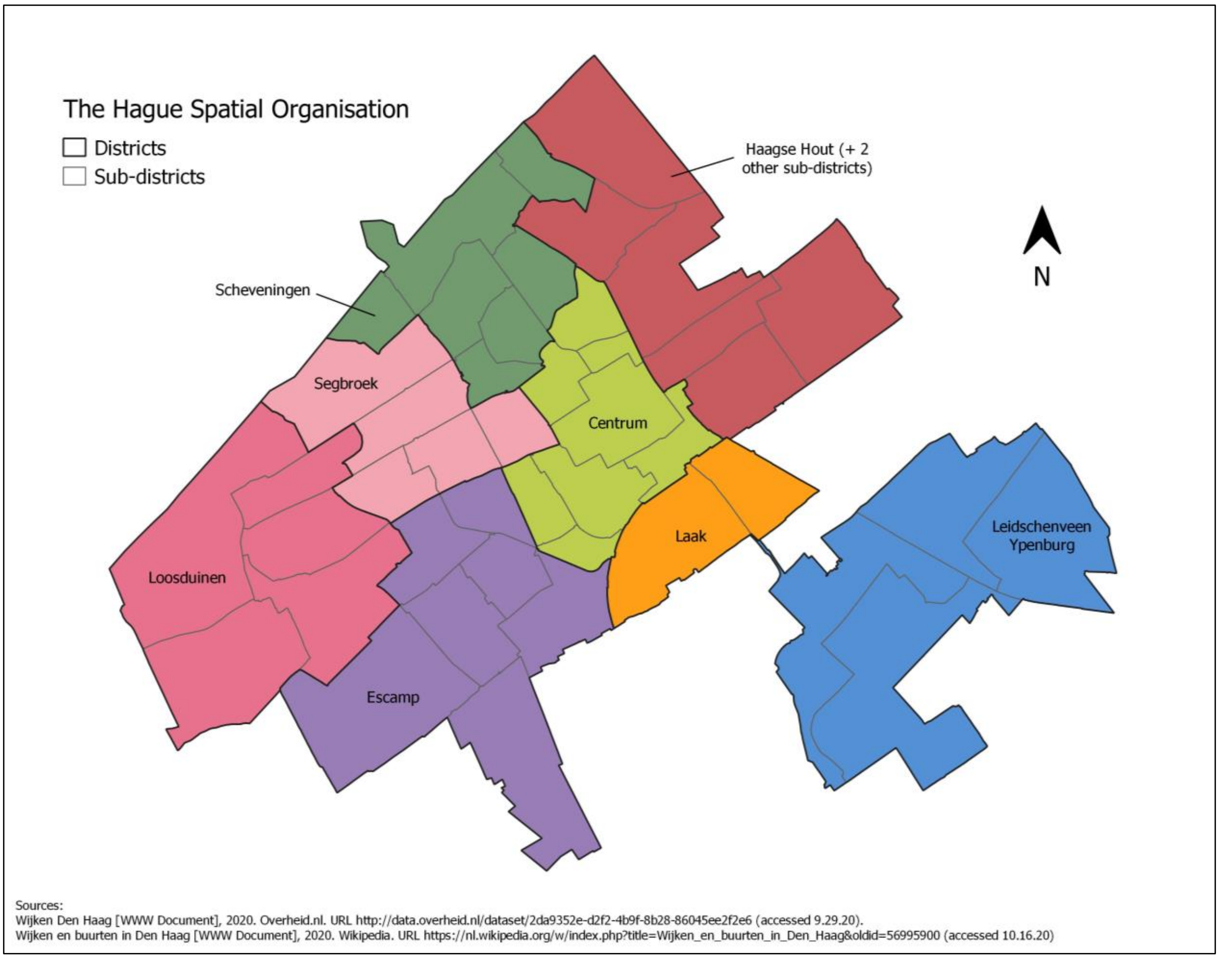

Figure S1 – The Hague districts.

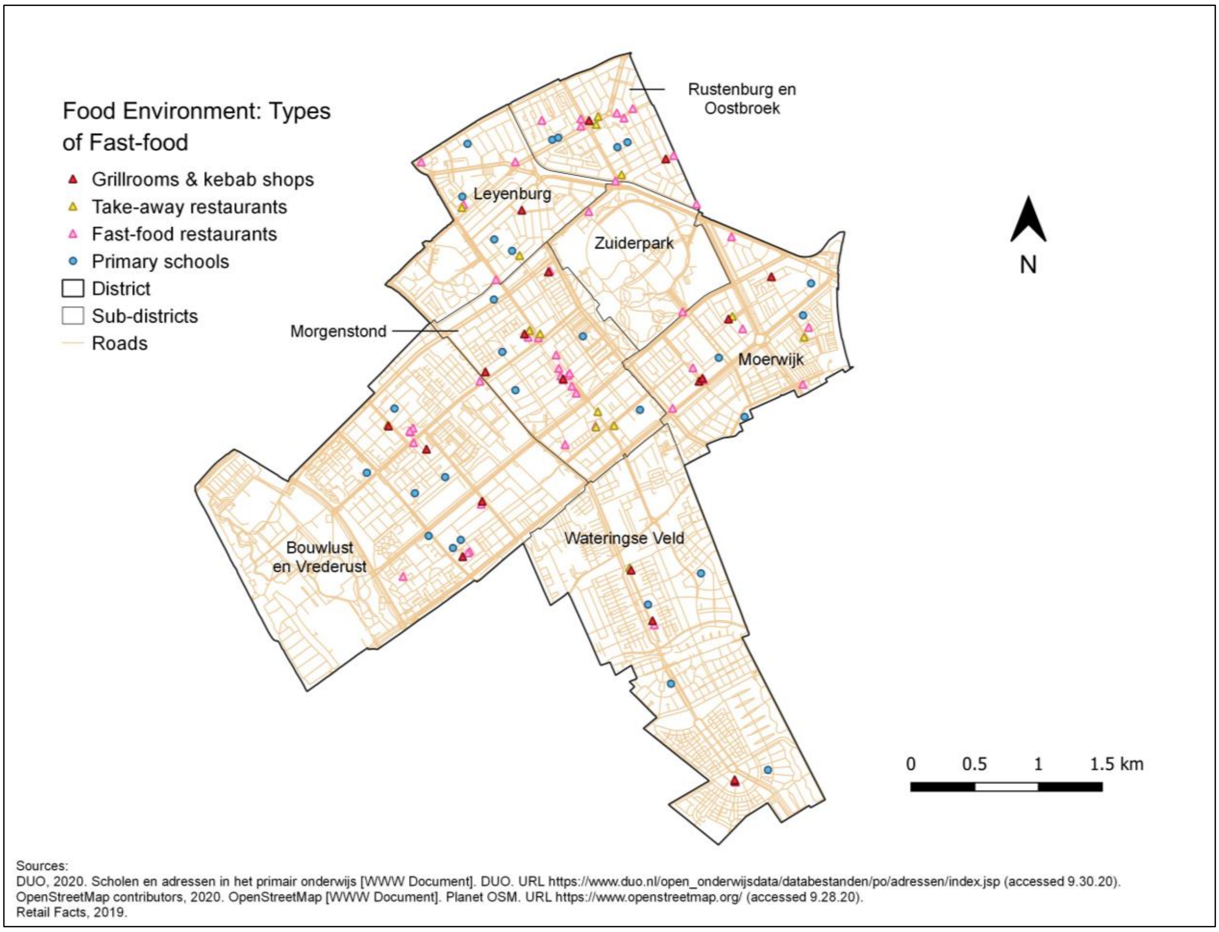

Figure S2 – Grillrooms and kebab shops, take-away restaurants and fast-food restaurants around primary schools in the district Escamp.

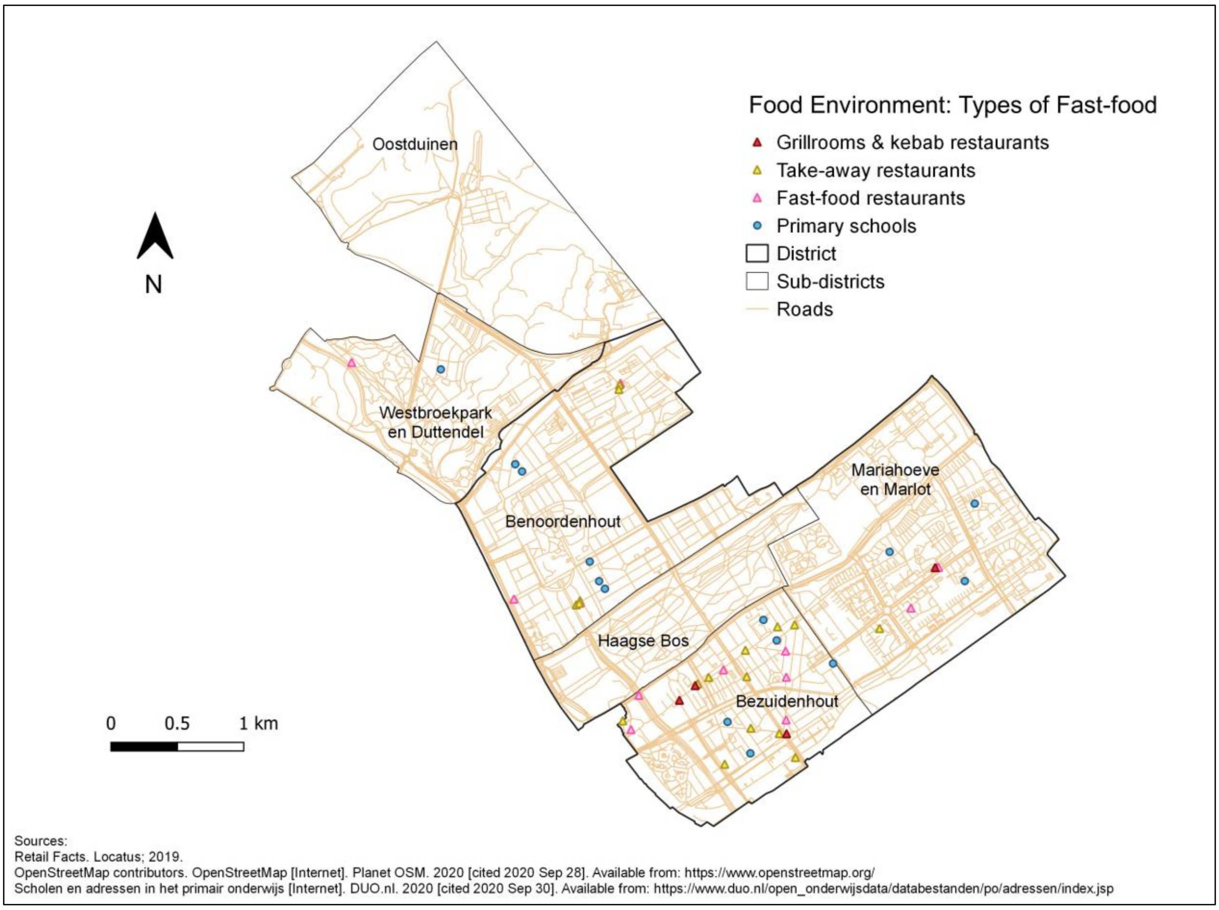

Figure S3 – Grillrooms and kebab shops, take-away restaurants and fast-food restaurants around primary schools in the district Haagse Hout and two adjacent sub-districts.

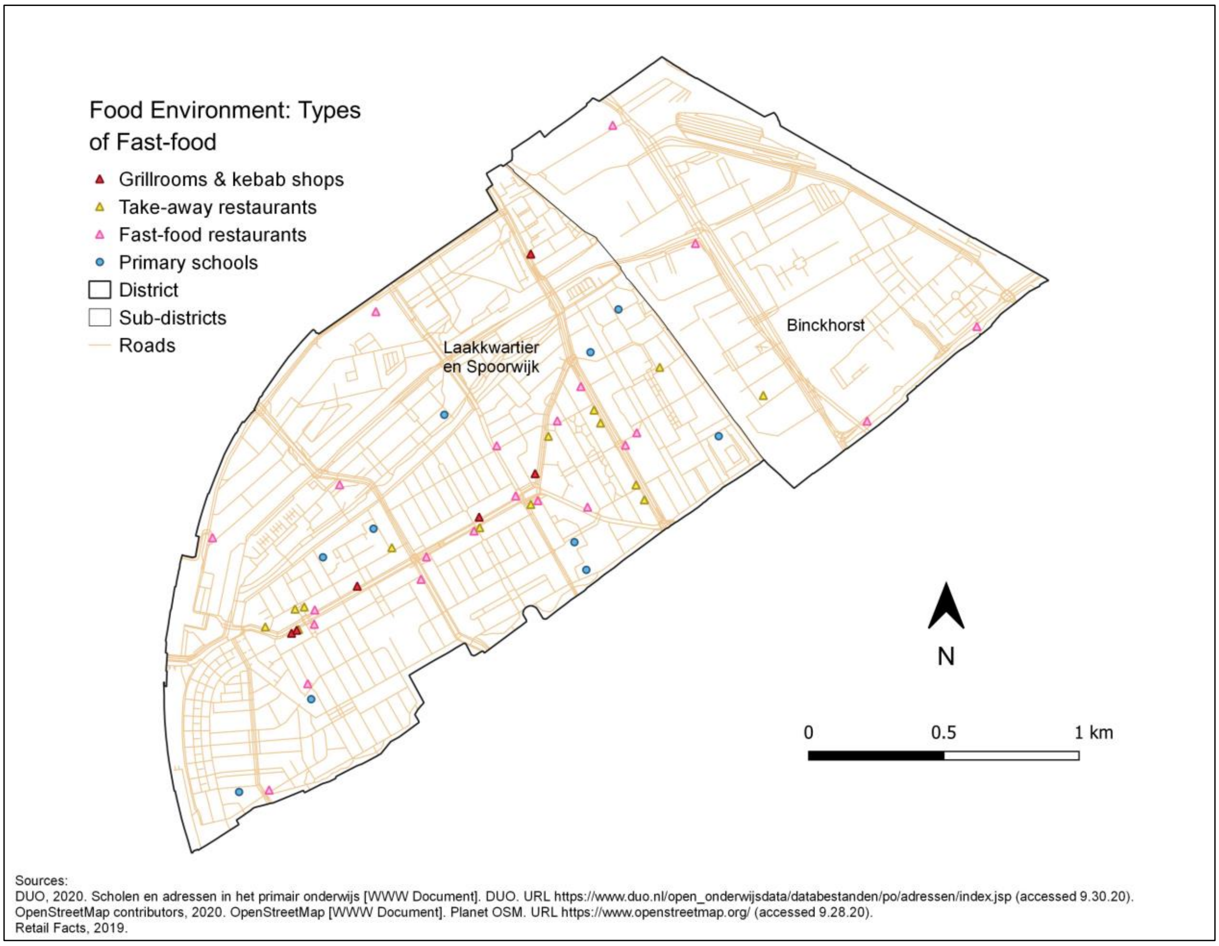

Figure S4 – Grillrooms and kebab shops, take-away restaurants and fast-food restaurants around primary schools in the district Laak.

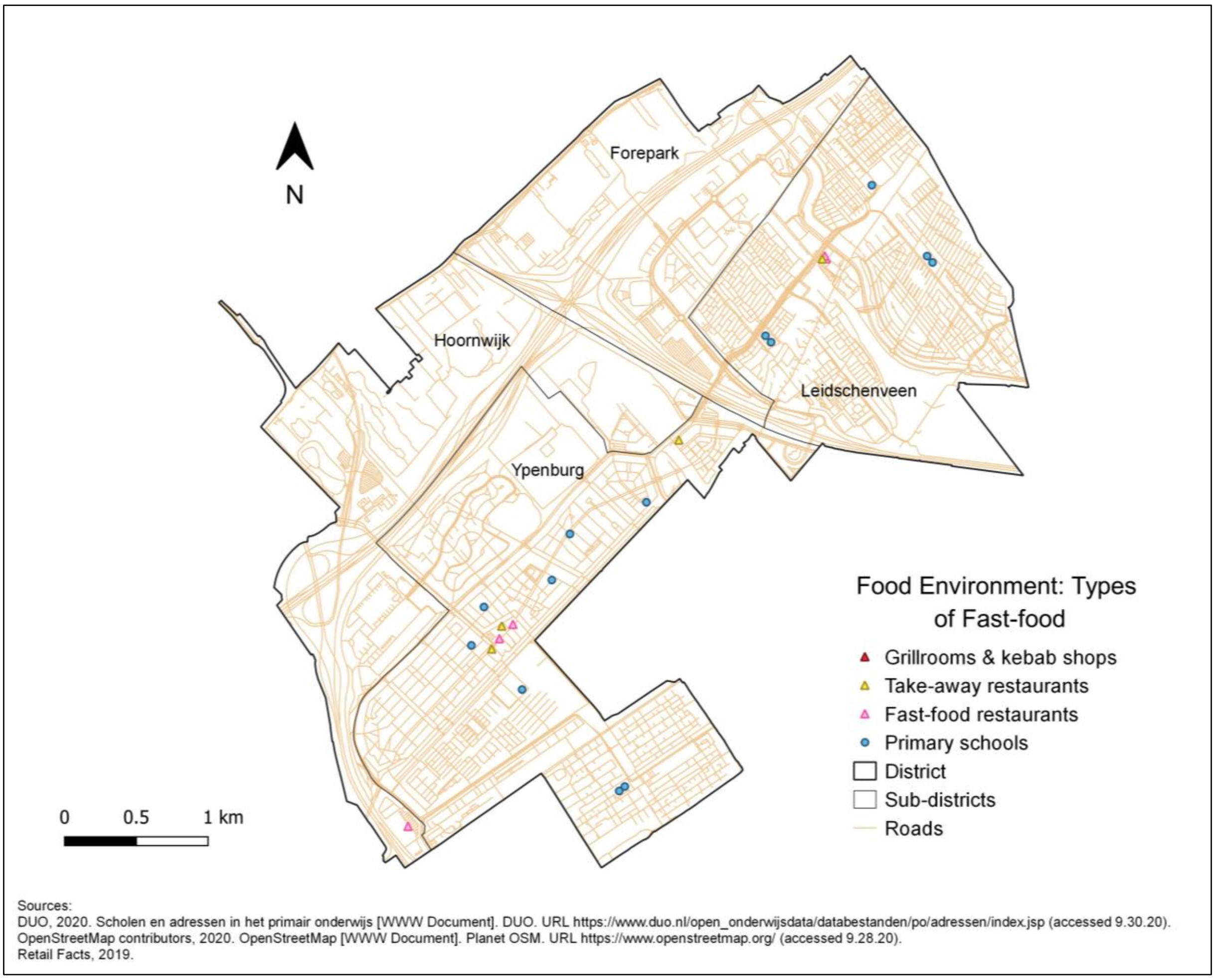

Figure S5 – Grillrooms and kebab shops, take-away restaurants and fast-food restaurants around primary schools in the district Leidschenveen-Ypenburg.

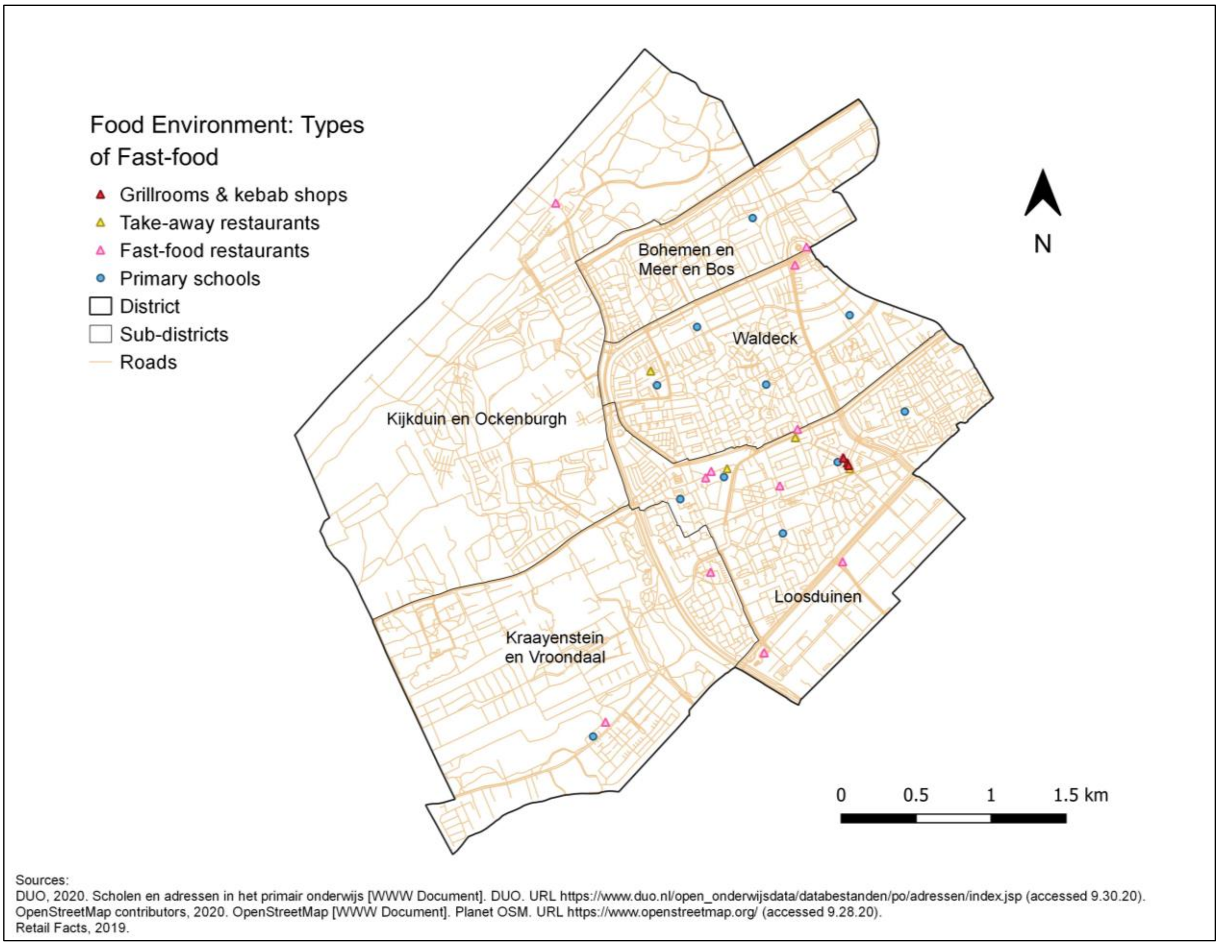

Figure S6 – Grillrooms and kebab shops, take-away restaurants and fast-food restaurants around primary schools in the district Loosduinen.

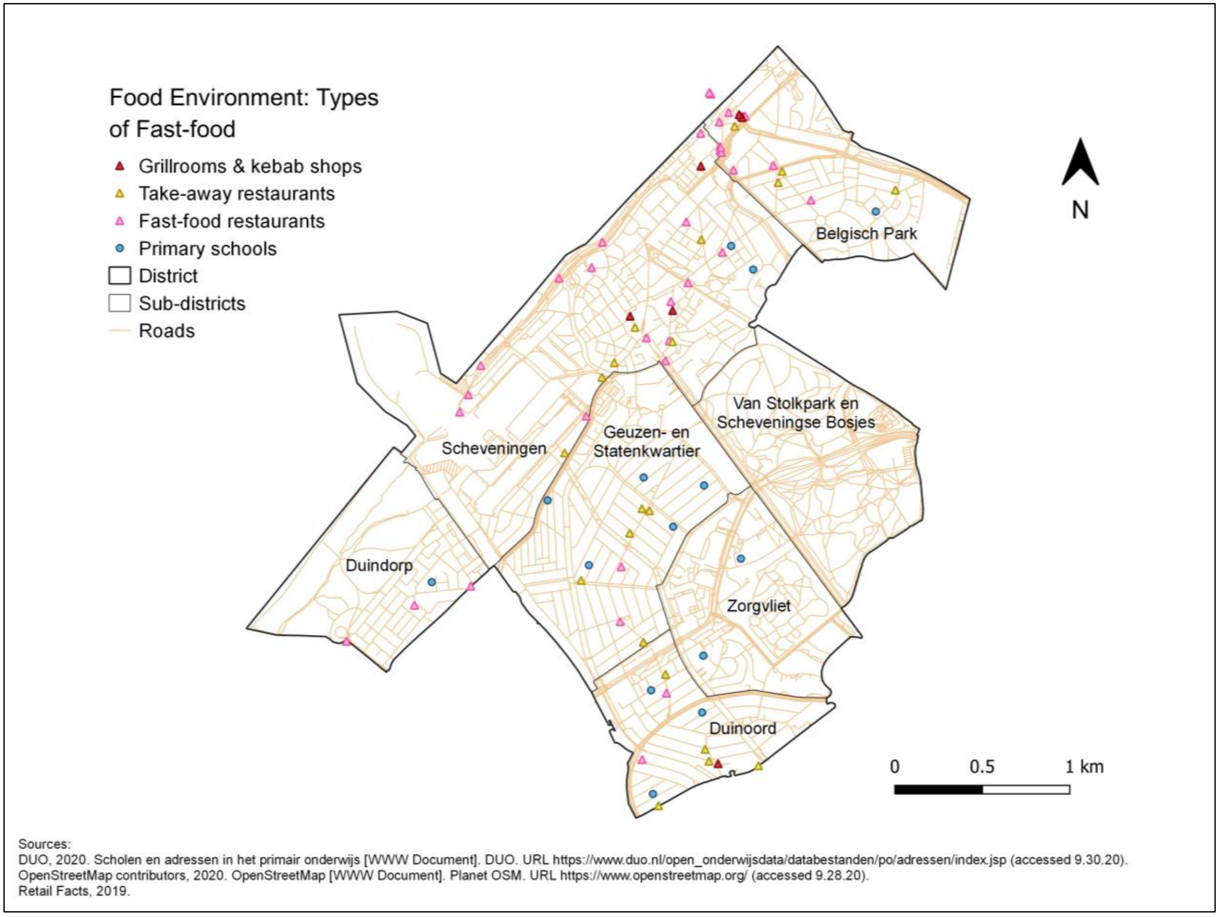

Figure S7 – Grillrooms and kebab shops, take-away restaurants and fast-food restaurants around primary schools in the district Scheveningen.

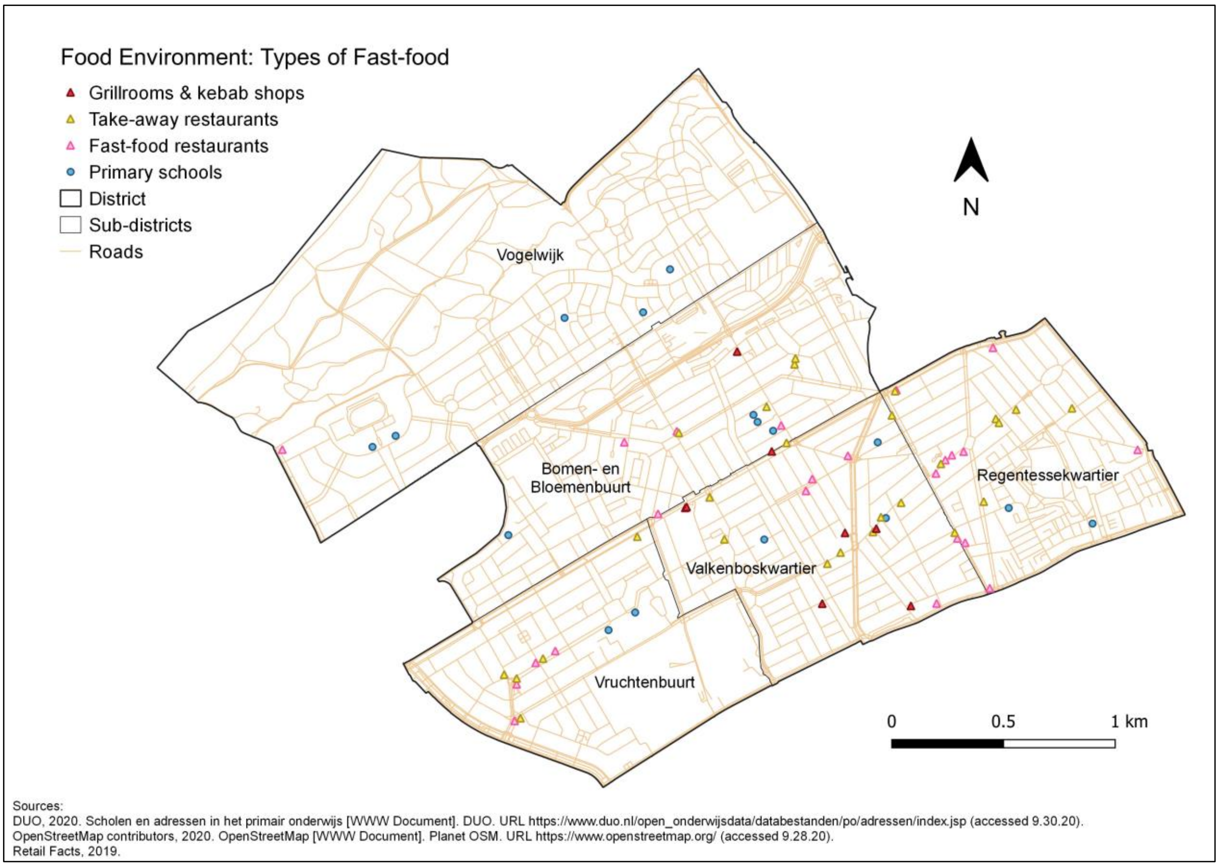

Figure S8 – Grillrooms and kebab shops, take-away restaurants and fast-food restaurants around primary schools in the district Segbroek.

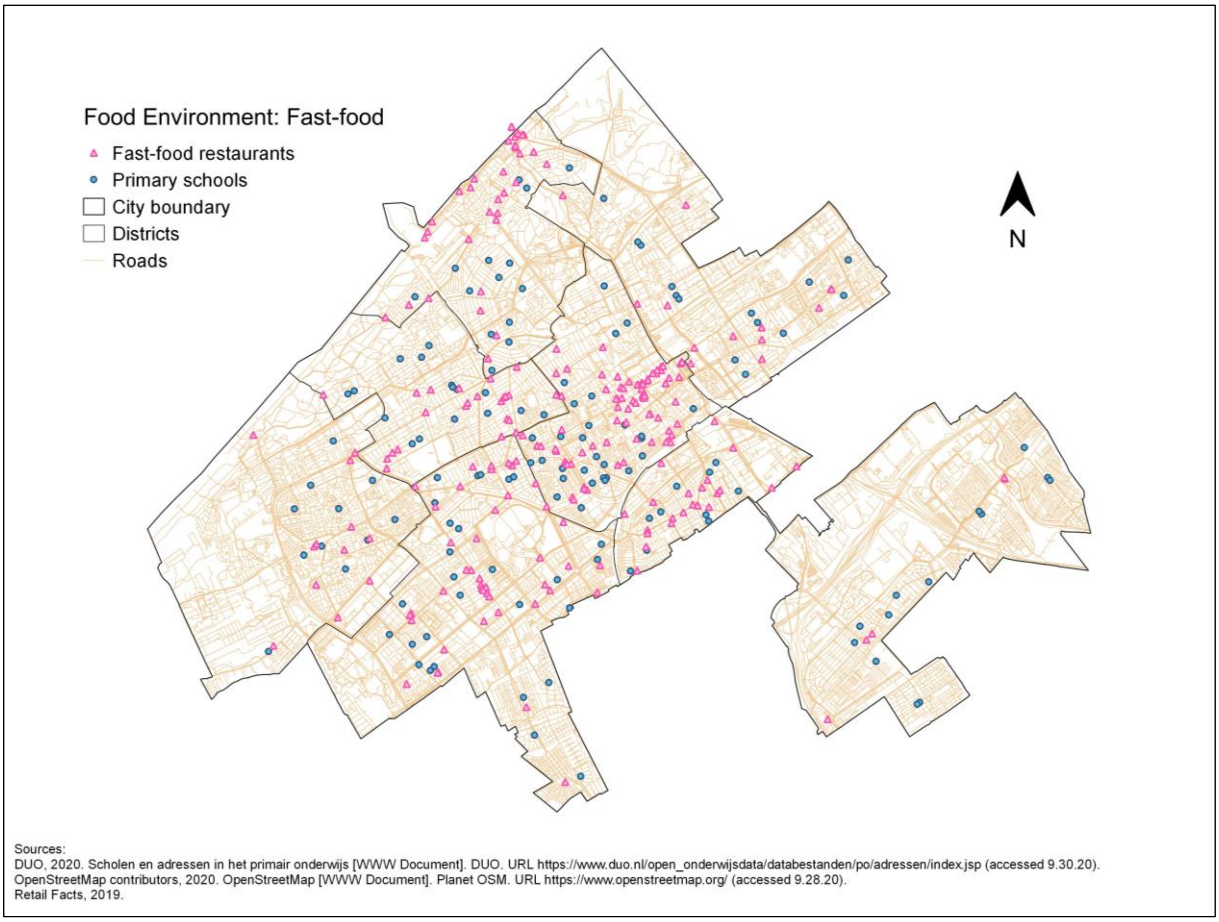

Figure S9 – Fast-food restaurants around primary schools in The Hague.

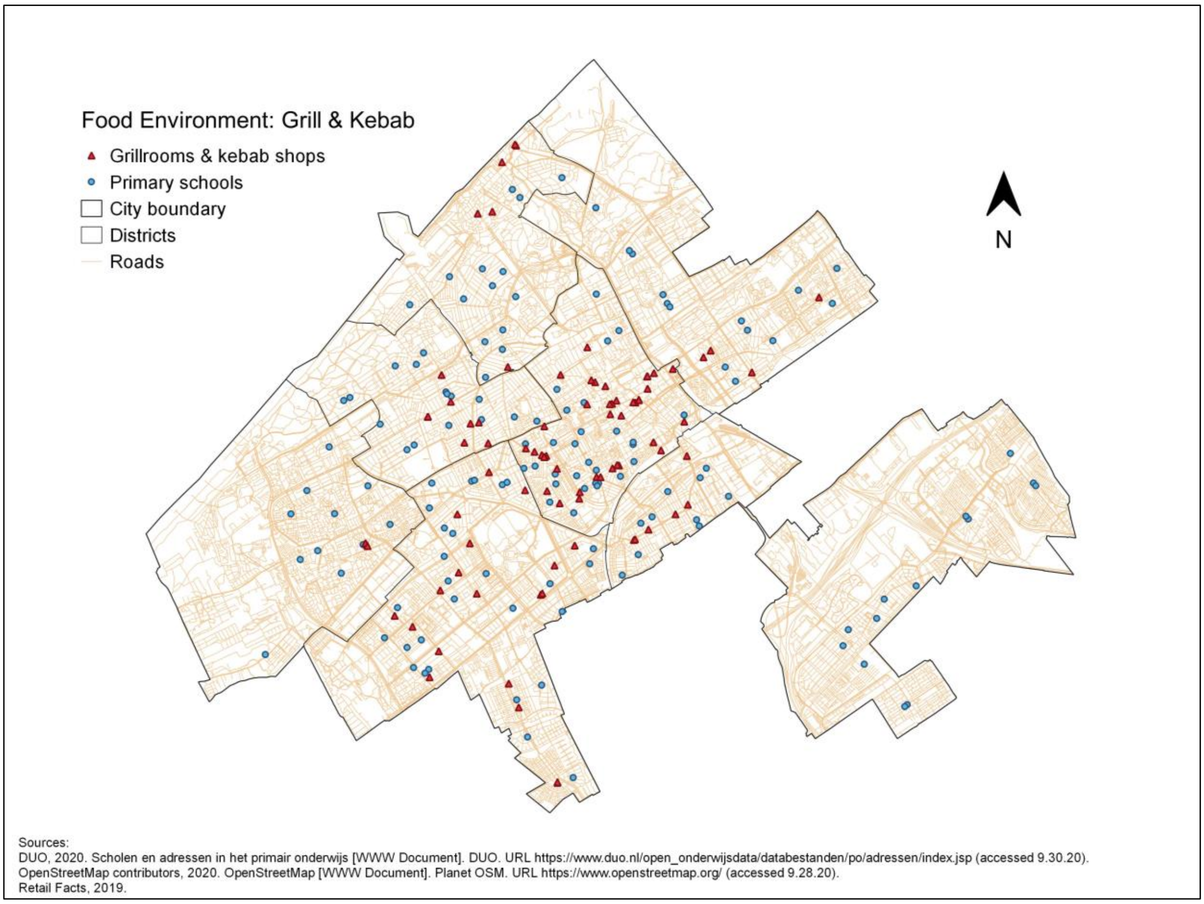

Figure S10 – Grillrooms and kebab shops around primary schools in The Hague.

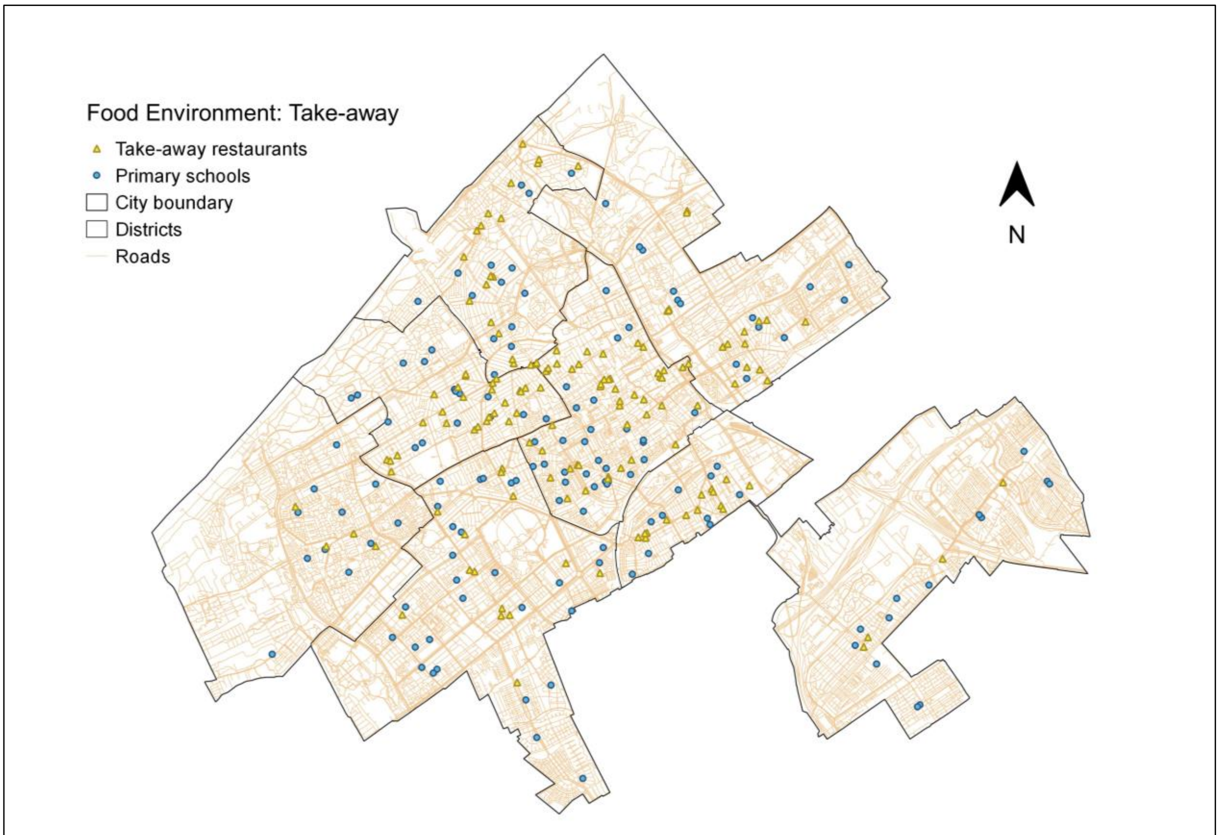

Sources:
DUO, 2020. Scholen en adressen in het primair onderwijs [WWW Document]. DUO. URL https://www.duo.nl/open_onderwijsdata/databestanden/po/adressen/index.jsp (accessed 9.30.20).
OpenStreetMap contributors, 2020. OpenStreetMap [WWW Document]. Planet OSM. URL https://www.openstreetmap.org/ (accessed 9.28.20).
Retail Facts, 2019.

Figure S11 – Take-away restaurants around primary schools in The Hague.
